# Supplementary material for: River-bed armouring as a granular segregation phenomenon
Source: Nat Commun. 2017 Nov 8;8:1363. doi: 10.1038/s41467-017-01681-3 (PMC5678076; doi:10.1038/s41467-017-01681-3)
Supplement: Supplementary file 1 — Supplementary Information [file 41467_2017_1681_MOESM1_ESM.pdf]

Supplementary Table 1. Parameters of the PDE simulations for five Shields numbers studied in the main manuscript.

Shields number, τ_s^*	β value	U_{sf} (m s^{-1})	$\langle v_x \rangle$ (m s^{-1})
$2.7\tau_{cs}^*$	2.30e1	7.0e-3	1.1e-3
$3.8\tau_{cs}^*$	1.85e1	1.5e-2	1.4e-3
$4.1\tau_{cs}^*$	1.30e1	6.0e-2	3.3e-3
$4.4\tau_{cs}^*$	1.25e1	8.5e-2	5.2e-3
$4.7\tau_{cs}^*$	1.20e1	9.0e-2	6.2e-3

Supplementary Table 2. Simulation parameters

Parameter	Value
Grain density, ρ	1190 kg m^{-3}
Grain diameters, d	[$d_s = 0.0015$, $d_l = 0.003$] m
Gravitational acceleration, $\vec{g}$	9.81 m s^{-2}
Young's modulus, E	5×10^6 N m^{-2}
Poisson ratio, ν	0.45
Friction coefficient, μ	0.5
Coefficient of restitution, e_n	0.01
Time step, Δt	2×10^{-6} s
Shear velocity, u_{top}	[0.05, 0.08] m s^{-1}

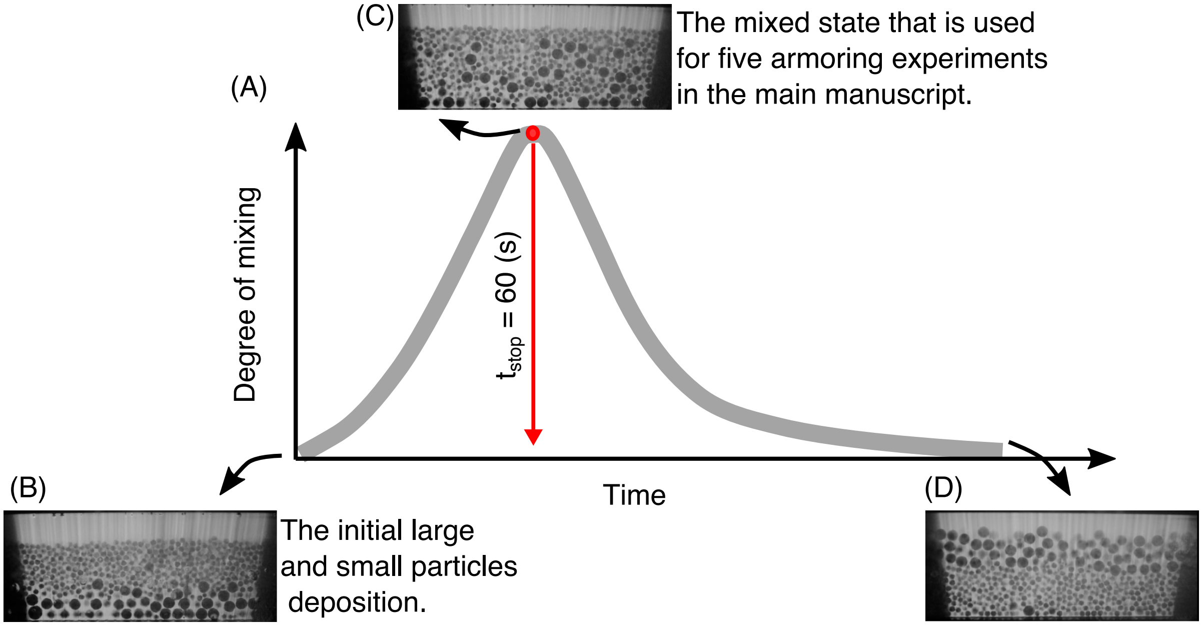

Supplementary Figure 1. (A) A schematic for the preparation protocol used for preparing the initial bidisperse mixture of particles. Small and large particles are initially deposited in an inversely segregated manner (panel B). The initial deposition has been performed by gently pouring first large particles and then small particles uniformly but manually from a very close distance to the bottom of the chamber. The system is then subjected to a rotation of $\Omega = 22\,\text{r.p.m.}$ that is a driving stress equivalent to $\tau_s^* = 20\tau_{cs}^*$ for ~ 1 minute in order to mix the large and small populations (panel C). This shear stress was sufficient to fully suspend all particles in the channel. Fluid shear was then stopped completely and the suspension was left for ~ 30 minutes to allow time for sedimentation, and relaxation and compaction of the granular bed, to reach completion. The final random packed layer at the end of preparation protocol has a thickness $\sim 15.5d_s$ for all experiments. This state is used for the five Shields number armoring experiments reported in the main manuscript. (D) A hypothetical fully segregated state that one could obtain if continuing shearing the initial sample at the large preparation shear stress of $\tau_s^* = 20\tau_{cs}^*$ for about 3 minutes.

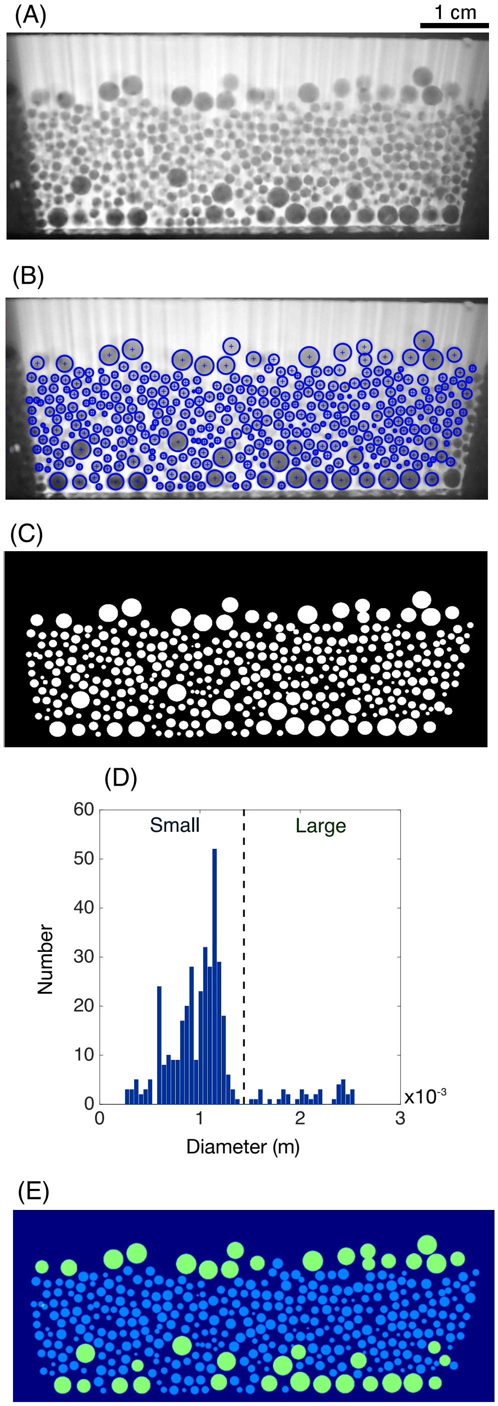

Supplementary Figure 2. (A) An example raw image from the experimental run. (B) Detected particles for panel (A) after processing of the image and running our particle detection algorithm. (C) Binary image of particles detected in panel (B). Time sequences of similar images are used for calculating streamwise velocity profiles using cross-correlation analysis. (D) Size distribution of detected particles, diameter threshold for small and large particles and the resulting subsets. (E) Detected small and large particles for the example snapshot in panel (A). This is the final result of the image analysis and particle detection, and similar images are used for all post-processing and further analysis presented in the main manuscript.

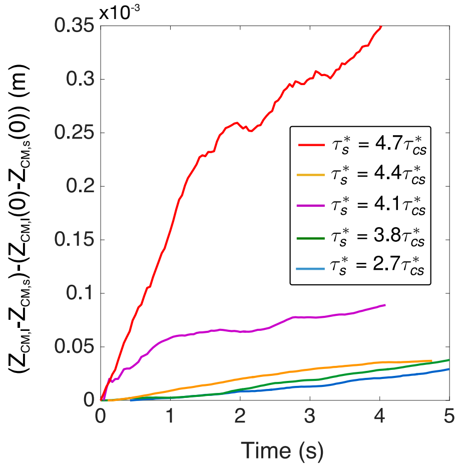

Supplementary Figure 3. Relative position of the vertical (z) component of the center of mass of the assembly of large and small particles at the start of the experimental runs for five Shields numbers. Here, $Z_{CM,l}$ and $Z_{CM,s}$ are the z component of the center of mass position of the assembly of large and small particles. $Z_{CM,l}(0)$ and $Z_{CM,s}(0)$ denote the initial positions of the center of masses of two assemblies, i.e. the positions at $t = 0$.

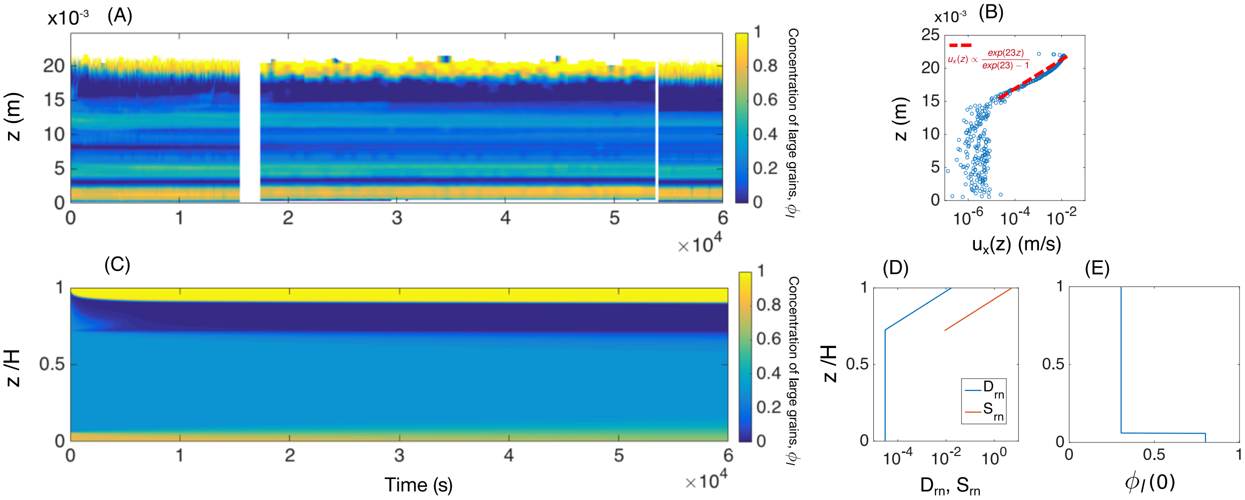

Supplementary Figure 4. (A) 1D (x-averaged) concentration map of large grains over time for shear stress $\tau_s^* = 2.7\tau_{cs}^*$. (B) Streamwise velocity profile ($u_x(z)$) of the granular bed for shear stress $\tau_s^* = 2.7\tau_{cs}^*$. (C) 1D ($x$-averaged) concentration map of large grains over time from the advection-diffusion model, with velocity profiles and initial condition corresponding to the shear stress $\tau_s^* = 2.7\tau_{cs}^*$ in panel (A). (D) Vertical profiles of S_{rn} and D_{rn} that were implemented in the continuum model. (E) Initial vertical profile of concentration of large grains ($\phi_l(t = 0)$)

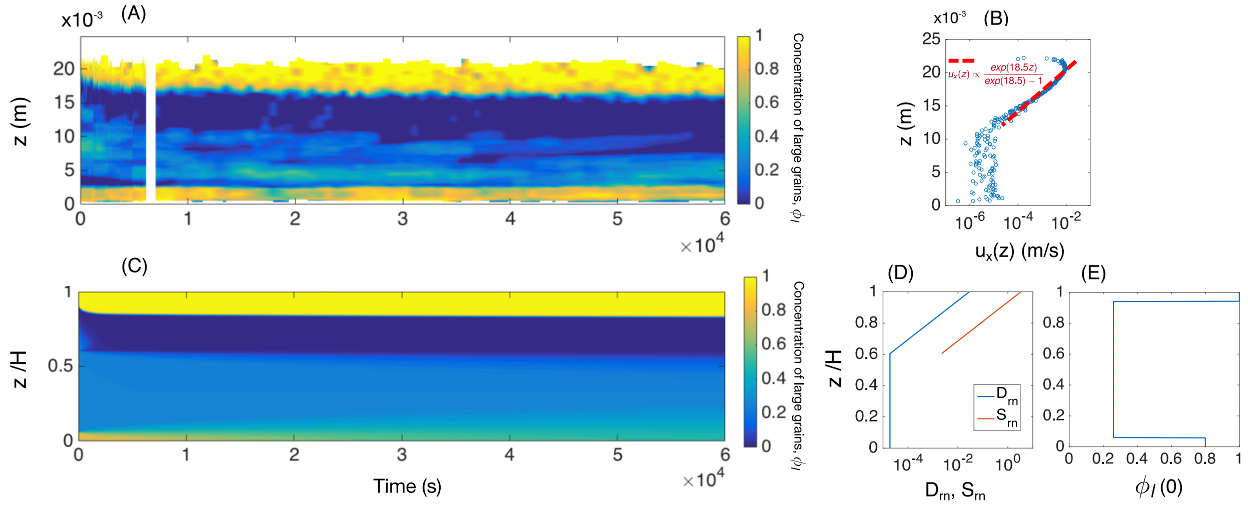

Supplementary Figure 5. Concentration map for large grains for $\tau_s^* = 3.8\tau_{cs}^*$ experiment. All panels follow Supplementary Figure 4.

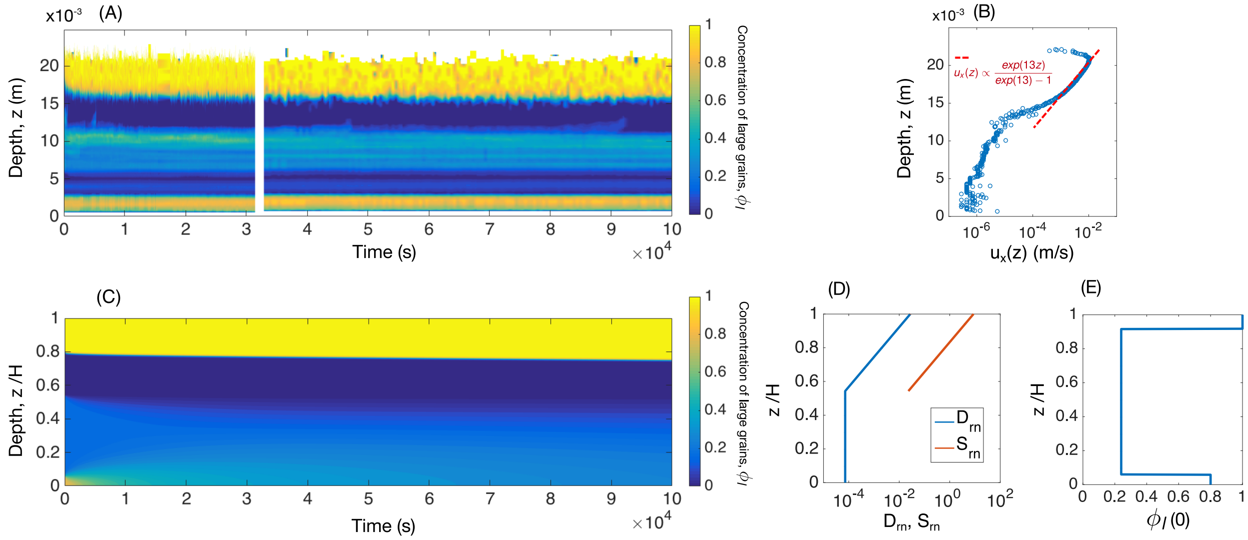

Supplementary Figure 6. Concentration map for large grains for $\tau_s^* = 4.1\tau_{cs}^*$ experiment. All panels follow Supplementary Figure 4.

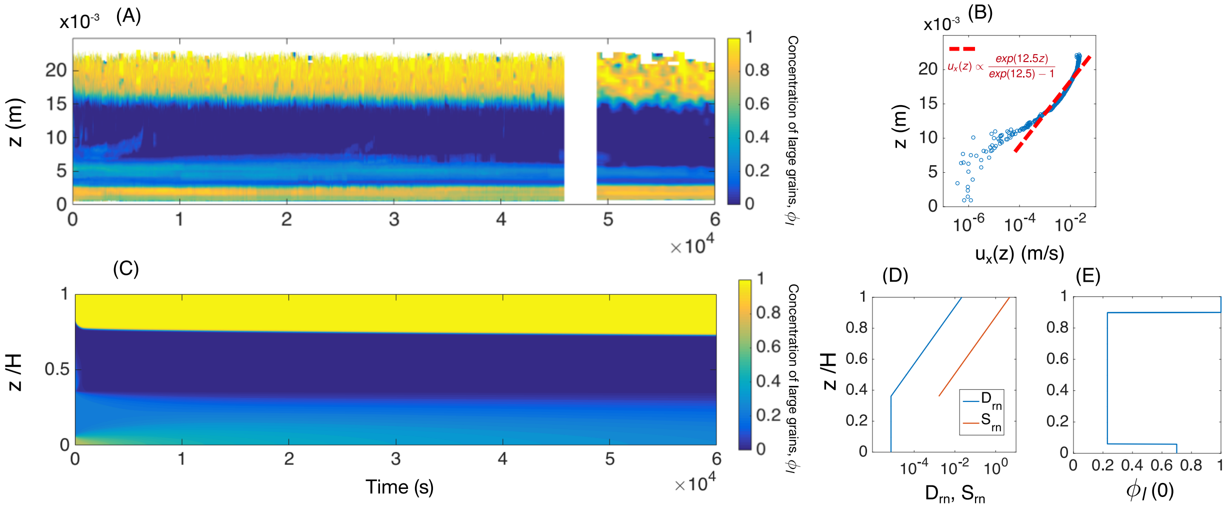

Supplementary Figure 7. Concentration map for large grains for $\tau_s^* = 4.4\tau_{cs}^*$ experiment. All panels follow Supplementary Figure 4.

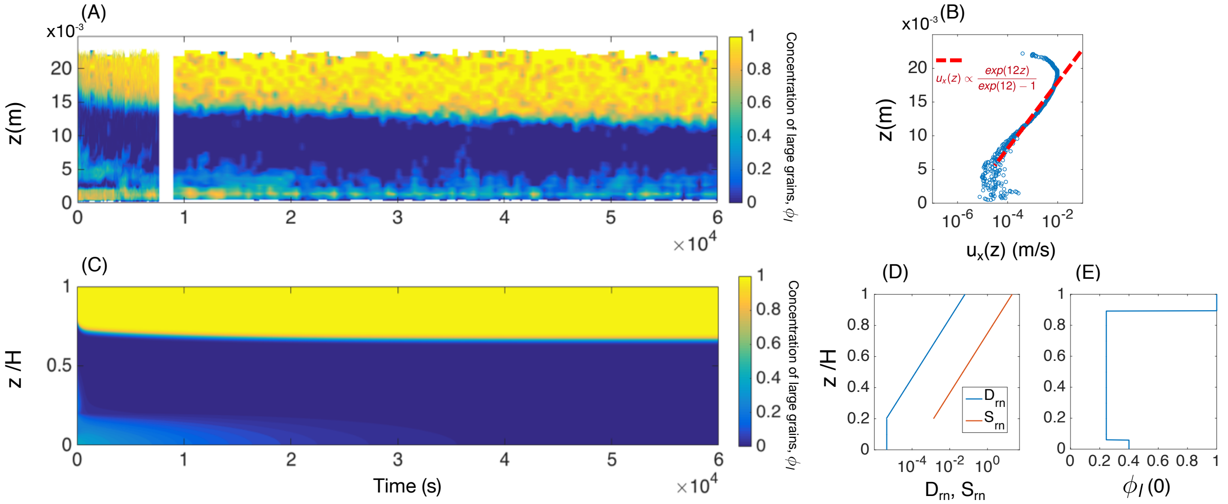

Supplementary Figure 8. Concentration map for large grains for $\tau_s^* = 4.7\tau_{cs}^*$ experiment. All panels follow Supplementary Figure 4.

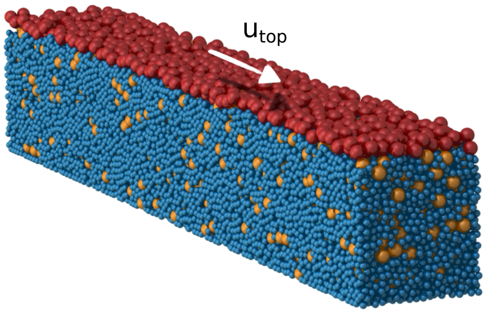

Supplementary Figure 9. A snapshot from the initial conditions of the numerical DEM simulation that shows the layer of large grains deposited at the surface and moving at constant velocity u_{top}.

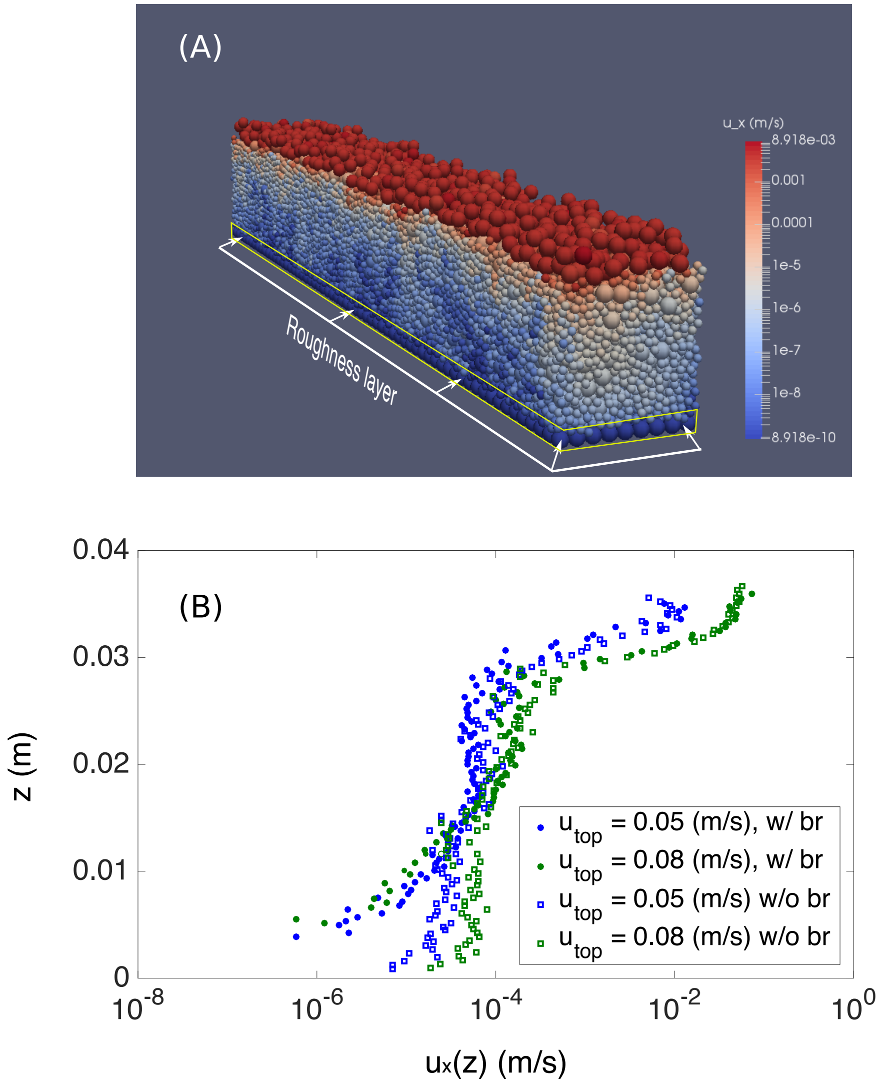

Supplementary Figure 10. (A) A snapshot from the armored/segregated state of a simulation with a roughness layer at its base. The simulation is run with $u_{top} = 0.08$ m s^{-1}. (B) Velocity profiles for simulations with ("w/ br" in legend) and without ("w/o br" in legend) the roughness layer, at two surface layer velocities, u_{top}, of 0.05 and 0.08 m s^{-1}.

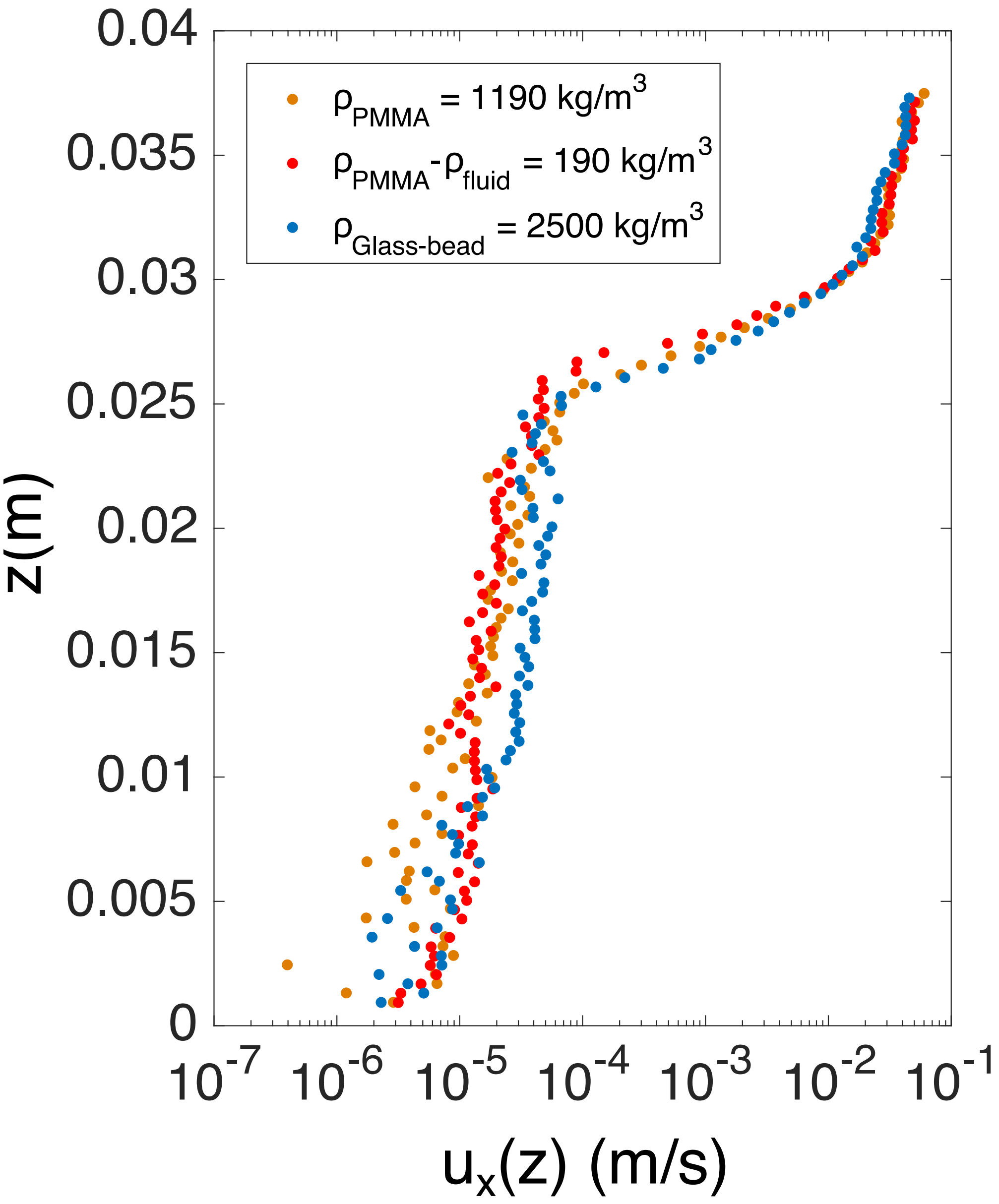

Supplementary Figure 11. Velocity profiles ($u_x(z)$) for DEM simulations of bidisperse sheared bed with different grain densities (ρ_{PMMA}, $\rho_{PMMA} - \rho_{fluid}$, and $\rho_{Glass-bead}$ for PMMA, submerged PMMA and glass bead, respectively). All systems are driven with surface layer velocity $u_{top} = 0.05$ m s^{-1}.
