# Supplementary material for: River-bed armouring as a granular segregation phenomenon
Source: Nat Commun. 2017 Nov 8;8:1363. doi: 10.1038/s41467-017-01681-3 (PMC5678076; doi:10.1038/s41467-017-01681-3)
Supplement: Supplementary file 3 — Description of Additional Supplementary Files [file 41467_2017_1681_MOESM3_ESM.pdf]

Description of Additional Supplementary Files

File Name: Supplementary Movie 1
Description: Real-time video of the first 30 seconds of the armoring experimental run at shear stress $\tau * s = 3.8\tau * cs$. The real duration of the video is 30 seconds, the same as its playback time.

File Name: Supplementary Movie 2
Description: Time-lapse video of the armoring experimental run at shear stress $\tau * s = 3.8\tau * cs$. The real duration of the video is 22.9 hrs, but its playback time is 8 seconds. Note that the snapshots are logarithmically spaced in time.
